# Organic amendments enhance Pb tolerance and accumulation during micropropagation of *Daphne jasminea*

**DOI:** 10.1007/s11356-016-7977-2

**Published:** 2016-11-05

**Authors:** Alina Wiszniewska, Ewa Muszyńska, Ewa Hanus-Fajerska, Sylwester Smoleń, Michał Dziurka, Kinga Dziurka

**Affiliations:** 1 Unit of Botany and Plant Physiology, Institute of Plant Biology and Biotechnology, Faculty of Biotechnology and Horticulture, University of Agriculture in Kraków, Al. 29 Listopada 54, 31-425 Kraków, Poland; 2 Department of Botany, Faculty of Agriculture and Biology, Warsaw University of Life Sciences (SGGW), Nowoursynowska 159, Building 37, 02-776 Warszawa, Poland; 3 Unit of Plant Nutrition, Institute of Plant Biology and Biotechnology, Faculty of Biotechnology and Horticulture, University of Agriculture in Kraków, Al. 29 Listopada 54, 31-425 Kraków, Poland; 4 The Franciszek Górski Institute of Plant Physiology, Polish Academy of Sciences, Niezapominajek 21, 30-239 Kraków, Poland

**Keywords:** Antioxidant activity, Biostimulation, In vitro culture, Lead adaptation, Medium supplements, Phenolic compounds, Thymelaeaceae

## Abstract

The study investigated the effects of organic amendments: pineapple pulp (PP) and agar hydrolyzate (AH), on micropropagation and Pb bioaccumulation and tolerance in a woody shrub *Daphne jasminea* cultured in vitro. The amendments were analyzed for their content of carbohydrates, phenolic acids, and phytohormones and added at a dose of 10 mL L^−1^ to the medium containing 1.0 mM lead nitrate. Micropropagation coefficient increased by 10.2–16.6 % in PP and AH variants, respectively. Growth tolerance index increased by 22.9–31.8 % for the shoots and by 60.1–82.4 % for the roots. In the absence of Pb, the additives inhibited multiplication and growth of microplantlets. PP and AH facilitated Pb accumulation in plant organs, especially in the roots. PP enhanced bioconcentration factor and AH improved Pb translocation to the shoots. Adaptation to Pb was associated with increased accumulation of phenolics and higher radical scavenging activity. Medium supplementation, particularly with AH, enhanced antiradical activity of Pb-adapted lines but reduced the content of phenolic compounds. The study results indicated that supplementation with organic amendments may be beneficial in in vitro selection against lead toxicity.

## Introduction

Lead (Pb) is one of the first metals discovered by the human race (Flora et al. 2012). Although it occurs naturally within the earth’s crust, its high concentrations in the environment result from anthropogenic activities. Majority of the emissions originate from metallurgy, mining, smelting, and combustion of coal. Moreover, due to the unique properties of lead, such as softness, high malleability, ductility, low melting point, and resistance to corrosion, it is widely used across different industries, as well as in agriculture as lead arsenate pesticide (Ciarkowska and Hanus-Fajerska 2008; Gupta et al. 2013; Ashraf et al. 2015). Its high toxicity and non-biodegradable nature make lead the second most hazardous toxin that poses a significant threat to all living organisms (Flora et al. 2012; Yuan et al. 2015). In vascular plants, lead affects morphophysiological and biochemical processes, such as seed germination and seedling growth, development of organs, and plant phenology (Shi et al. 2011; Muszyńska et al. 2013; Babu et al. 2014). Enhanced and uncontrolled production of reactive oxygen species (ROS) is another consequence of plant tissue exposure to lead ions. To counteract the injuries caused by oxidative stress, cells are equipped with defense mechanisms that work by scavenging excessive ROS. Apart from antioxidant enzymes, the antioxidant system in Pb-treated plants involves non-enzymatic scavengers like phenolic compounds, glutathione, and organic acids (Michalak 2006; Gill and Tuteja 2010; Sharma et al. 2012).

In in vitro cultures, plant cells and organs may be screened for their tolerance to elevated concentrations of heavy metals (Ghnaya et al. 2010; Di Lonardo et al. 2011; Bernabe-Antonio et al. 2015; Wiszniewska et al. 2015). Advantages of in vitro selection include controlled culture environment, particularly with regard to a medium composition and the possibility of testing the effects of medium additives in the context of metal toxicity (Doran 2009). Medium supplements may help to unveil the mechanisms of metal tolerance and also serve as a source of nutrients and bioactive compounds that improve plant growth conditions under heavy metal stress. Additional supply of carbon, growth regulators, and signaling molecules from organic products was reported to increase multiplication rate and formation of intact healthy plantlets (Bois 1992; Neumann et al. 2009; Wiszniewska et al. 2013; Gayathri et al. 2015). The use of organic amendments may also facilitate the studies on phytoremediation. An emerging technology called assisted (aided, enhanced) phytoremediation seeks to improve soil clean-up by manipulating the growing conditions of the remediating plants (Tack and Meers 2010). Nowadays, numerous inorganic and organic amendments, mainly municipal and agrowastes, are used in assisted phytoremediation of polluted soils (Bolan and Duraisamy 2003; Park et al. 2011). An interesting alternative to this is an exploitation of natural products, such as microbial exudates and plant extracts (Wang et al. 2011b; Stingu et al. 2012; Li et al. 2015). The advantages of using natural organic supplements in remedial work include their low toxicity and high biodegradability. Kuppusamy et al. (2015) discussed also the unrevealed potential of polyphenols, present in almost all plant-derived materials, as growth stimulators during phyto/rhizoremediation. However, there are no literature data on using medium supplements to stimulate the growth of plants selected in vitro in the presence of lead. Recently, we have obtained vigorous, proliferative shoot cultures of a woody shrub, *Daphne jasminea* (Thymelaeaceae), in the course of in vitro selection on lead-containing media (Wiszniewska et al. 2015). Ornamental features of *D. jasminea* together with its ability to grow in the presence of lead ions make this species interesting for exploitation in urban environment, threatened with heavy metal contamination, provided that rooting of the shoots can be achieved. Therefore, the aim of this study was to find out whether the addition of two organic supplements, i.e., pineapple pulp and agar hydrolyzate, to the culture medium would improve multiplication of *D. jasminea* shoots and plantlet formation during in vitro selection toward elevated tolerance to lead ions. Additionally, the effects of these additives on Pb^+2^ tolerance, accumulation, and transportation in the developing organs were studied. Pineapple pulp has recently been investigated in in vitro cultures of *Daphne* sp., and it was reported to promote shoot and root development (Wiszniewska et al. 2013). Agar hydrolyzate may be a source of compounds that play a regulatory role in organogenesis, i.e., oligosaccharins (short fragments of hemicelluloses) (Bois 1992), and phytohormone-like substances (Arthur et al. 2004). In the course of the experiments, we have successfully established Pb-tolerant shoot culture lines of *D. jasminea* and in this work, we intended to compare specific elements of biochemical response to Pb and organic supplements between plantlets adapted and non-adapted to lead.

## Materials and methods

### Plant material

Stock cultures of *D. jasminea* (Sibth. & Sm.) shoots were maintained on basal WPM medium (Lloyd and McCown 1981), containing MS vitamins (Murashige and Skoog 1962), 12.3 μM N6-[2-isopentyl] adenine (2iP), 5.37 μM 1-naphthaleneacetic acid (NAA), 0.5 g L^−1^ polyvinylpyrrolidone (PVP), 0.5 g L^−1^ 2-N-morpholino-ethanesulfonic acid (MES), 0.6 g L^−1^ activated charcoal, 0.65 g L^−1^ calcium gluconate, and 20.0 g L^−1^ sucrose, and solidified with 0.8 % Difco agar. The medium pH was adjusted to 5.6.

### In vitro culture conditions

Test cultures were established by placing 5-mm long explants on modified basal media containing lead nitrate and one of the tested supplements: (i) 1.0 mM Pb(NO_3_)_2_ and 10 mL L^−1^ of pineapple pulp (PP) and (ii) 1.0 mM Pb(NO_3_)_2_ and 10 mL L^−1^ of agar hydrolyzate (AH). Control treatments were the media containing lead nitrate only: (iii) 1.0 mM Pb(NO_3_)_2_ and tested supplement only: (iv) 10 mL L^−1^ of pineapple pulp; (v) 10 mL L^−1^ of agar hydrolyzate, as well as (vi) basal medium containing neither lead nor the supplement (see also Table 3). All media were prepared directly before the culture establishment and autoclaved at 121 °C, 0.1 MPa for 15 min. Both lead nitrate and organic supplements were added prior to autoclaving. The medium pH was adjusted to 5.6.

Ten microcuttings per 250-mL Erlenmeyer flask were explanted on the respective media. The cultures were maintained for 16 weeks in a growth chamber at 24 °C, under 16-h photoperiod (irradiance 80 μmol m^−2^ s^−1^), with one subculture after 8 weeks.

### Production of medium supplements

#### Pineapple pulp

The pineapple pulp was produced according to Kitsaki et al. (2004). Shortly, five ripe pineapples were homogenized using a blender. The pulp (~660 mL) was filtered through a cheesecloth, deproteinized by boiling for 10 min, and stored in small batches in 1.5-mL microcentrifuge tubes at −20 °C.

#### Agar hydrolyzate

The agar hydrolyzate was produced according to Bois (1992). Shortly, 0.05 % hemicellulase (Sigma) was added to 1 L of 1.0 % Difco agar solution. pH was adjusted to 5.5 and the solution was incubated for 2 h at 50 °C with constant stirring. Afterward, the mixture was centrifuged for 15 min at 10,000×*g* and the supernatant (~870 mL) was boiled for 20 min to deactivate enzymatic proteins. The agar hydrolyzate was stored in small batches in 10-mL centrifuge tubes at −20 °C.

### Characterization of medium supplements

Soluble sugars were extracted and analyzed as reported by Pociecha and Dziurka (2015) with modifications, using HPLC Agilent 1200 system equipped with a degasser, a binary pump, an automated liquid sampler, and a thermostated column compartment (Agilent, Germany) and ESA Coulochem II electrochemical detector with 5040 Analytical Cell (ESA, USA) with an analog-to-digital converter. Phenolic acids were extracted and analyzed with an ultrahigh performance liquid chromatography (UHPLC) system (Agilent Infinity 1260) equipped with a binary pump, an autosampler, and a fluorescence detector (FLD). The method for phytohormone extraction and quantification was a modification of that published by Żur et al. (2015). The samples were analyzed with UHPLC (Agilent Infinity 1260, Agilent, Germany), coupled to a triple quadruple mass spectrometer (6410 Triple Quad LC/MS, Agilent, USA) equipped with electrospray ionization (ESI).

### Evaluation of plant growth parameters

After 16 weeks, the shoots were counted and micropropagation coefficient was calculated using the following formula:

$$ MC=\left(\mathrm{number}\ \mathrm{of}\ \mathrm{induced}\ \mathrm{adventitious}\ \mathrm{shoots}/\mathrm{total}\ \mathrm{number}\ \mathrm{of}\ \mathrm{explants}\right) $$

Shoots and roots were measured and weighted. For dry matter determination, the plant material was dried at 105 °C in an oven for 24 h and weighted afterward.

Growth tolerance index (in %) was calculated on the basis of dry weight of shoots and roots, using the formula:

$$ {GTI}_{\mathrm{S}}=\left(\mathrm{mean}\ \mathrm{dry}\ \mathrm{weight}\ \mathrm{of}\ \mathrm{shoots}\ \mathrm{developed}\ \mathrm{on}\ Pb-\mathrm{supplemented}\ \mathrm{media}/\mathrm{mean}\ \mathrm{dry}\ \mathrm{weight}\ \mathrm{of}\ \mathrm{shoots}\ \mathrm{developed}\ \mathrm{on}\ Pb-\mathrm{free}\ \mathrm{medium}\right)\times 100 $$

$$ {GTI}_{\mathrm{R}}=\left(\mathrm{mean}\ \mathrm{dry}\ \mathrm{weight}\ \mathrm{of}\ \mathrm{roots}\ \mathrm{developed}\ \mathrm{on}\ Pb-\mathrm{supplemented}\ \mathrm{media}/\mathrm{mean}\ \mathrm{dry}\ \mathrm{weight}\ \mathrm{of}\ \mathrm{roots}\ \mathrm{developed}\ \mathrm{on}\ Pb-\mathrm{free}\ \mathrm{medium}\right)\times 100 $$

### Determination of Pb content and accumulation factors

The content of Pb was determined using the inductively coupled plasma optical emission spectrometry (ICP-OES) technique with the use of a Prodigy Teledyne (Leeman Labs, USA) ICP-OES spectrometer.

Pb content was analyzed after sample digestion in nitric acid only (Pasławski and Migaszewski 2006). Samples of air-dried tissues were digested at 200 °C (15 min of warming followed by 15 min at the set temperature) in 10 mL 65 % super-pure HNO_3_ (Merck, Whitehouse, Station, NJ, USA) using a CEM MARS-5 Xpress Microwave system (CEM World Headquarters, Matthews, NC, USA). Digested samples were transferred quantitatively to the final volume of 25 mL using double-distilled water and analyzed. The same procedure was applied to determine the Pb content in the fresh medium.

The bioconcentration factor (BCF) and translocation factor (TF) for lead were calculated as follows:

$$ BCF=\mathrm{lead}\ \mathrm{concentration}\ \mathrm{in}\ \mathrm{microplantlets}\ \left(\mathrm{mg}\cdotp {\mathrm{kg}}^{-1}\right)/\mathrm{lead}\ \mathrm{concentration}\ \mathrm{in}\ \mathrm{culture}\ \mathrm{medium}\ \left(\mathrm{mg}\cdotp {\mathrm{kg}}^{-1}\right) $$

$$ TF=\mathrm{lead}\ \mathrm{concentration}\ \mathrm{in}\ \mathrm{shoots}\ \left(\mathrm{mg}\cdotp {\mathrm{kg}}^{-1}\right)/\mathrm{lead}\ \mathrm{concentration}\ \mathrm{in}\ \mathrm{roots}\ \left(\mathrm{mg}\cdotp {\mathrm{kg}}^{-1}\right) $$

### Establishment of a long-term culture (LT *Daphne* line)

Long-term culture (LT line) was initiated using 5-mm long microcuttings derived from shoots multiplicated on the medium supplemented with 1.0 mM Pb(NO_3_)_2_. The culture was maintained for 52 weeks (1 year) with regular passages onto the medium containing 1.0 mM Pb(NO_3_)_2_. Pb-adapted microplantlets were then used as a material for the experiment, performed as described above, on the media supplemented with both Pb and the organic product. In a reference culture (LN line), normal, non-Pb-adapted microshoots were also grown under the same experimental scheme.

### Biochemical analyses

#### Phenolic profile

Phenolic compounds (total phenols, phenolic acids, flavonols, and anthocyanins) were determined using UV/VIS spectrophotometry (Fukumoto and Mazza 2000). Chlorogenic acid (CGA), caffeic acid (CA), and quercetin (QC) were used as standards for total phenolic content (TPC), phenolic acids, and flavonols, respectively. Anthocyanin content was expressed as the cyanidin (CY), according to its molar extinction. Plant tissue (about 500 mg) was ground with 10 mL of 80 % methanol and centrifuged for 15 min at 4000 rpm. The supernatant was mixed with 0.1 % HCl (in 96 % ethanol) and 2 % HCl (in water), and after 15 min, the absorbance at 280, 320, 360, and 520 nm was read (Hitachi U-2900 spectrophotometer, Japan). The content of phenolic compounds was expressed in milligram of the respective standard equivalents per 100 g of fresh weight.

#### Radical scavenging activity

Stable free radical 2,2-diphenyl-1-picrylhydrazyl (DPPH) was used to test radical scavenging activity of *D. jasminea* organs (separately shoots and roots) (Pekkarinen et al. 1999). The changes in absorbance of DPPH solution, following reduction of DPPH, were measured at 517 nm at the time of the extract addition and after 30 min, using Hitachi U-2900 spectrophotometer. For the analysis, 80 % methanol extracts were used. The antioxidant activity of the extracts was expressed in percent of DPPH radical reduced by a unit of the plant extract.

### Experimental design and statistical analysis

The experiment was repeated independently 3 times (three replications), with at least 30 explants (microcuttings) per treatment within 1 replication. Microcuttings were randomly assigned to treatments. For biochemical analyses, minimum of 3 randomly chosen samples per treatment were used. The data were subjected to ANOVA analysis (STATISTICA 10.0, StatSoft, Tulsa, OK, USA), and a post hoc Duncan’s test was used to determine differences between treatments at *P* < 0.05.

## Results

### Characterization of medium supplements

Both organic supplements applied in this study were analyzed for the presence of sugars, phenolic acids, and phytohormones (Tables 1 and 2). Agar hydrolyzate (AH) contained 588.1 μg mL^−1^ of sugars, predominantly sucrose but also maltose, fructose, kestose, and glucose were detected (Table 1). Phenolic acids were present in very low concentrations of about 7 ng mL^−1^, and they were mainly ferulic and benzoic acid (Table 1). Among phytohormones, both auxins and cytokinins were detected (Table 1). Total concentration of cytokinins (37.6 pg mL^−1^) was two times higher than of auxins (18 pg mL^−1^). The most abundant phytohormones were cytokinin cis-zeatin riboside (21.9 pg mL^−1^) and auxin indole butyric acid (IBA) (16.2 pg mL^−1^). Abscisic acid was not detected in AH.

**Table 1:** Carbohydrates, phenolic acids, and phytohormones determined in agar hydrolyzate

Carbohydrates			
	μg/mL		μg/mL
Fructose	22.6	Kestose	10.1
Glucose	8.0	Isomaltotriose	–
Maltose	39.1	Nystose	–
Sucrose	508.4	Trehalose	–
Phenolic acids			
	ng/mL		ng/mL
Homovanillic acid	–	Ferulic acid	2.0
Vanillic acid	–	*p*-hydroxobenzoic acid	0.05
Cinnamic acid	–	Rosmarinic acid	–
Syringic acid	0.02	Chlorogenic acid	2
Sinapic acid	0.2	Gallic acid	–
Caffeic acid	–	3,4-dihydroxobenzoic acid	0.2
Benzoic acid	1.8	salicylic acid	0.1
Coumaric acid	0.5	Gentisic acid	0.1
Phytohormones			
	pg/mL		pg/mL
t-zeatin	–	N6-(2-isopentenyl) adenine	6.7
c-zeatin	–	Kinetin riboside	8.2
kinetin	0.8	Indole-3-acetic acid	1.8
t-zeatin riboside	–	Abscisic acid	–
c-zeatin riboside	21.9	Indole-3-butyric acid	16.2

**Table 2:** Carbohydrates, phenolic acids, and phytohormones determined in pineapple pulp

Carbohydrates			
	μg/mL		μg/mL
Fructose	16,939.0	Kestose	420.5
Glucose	16,920.7	Isomaltotriose	131.2
Maltose	1610.7	Nystose	6.3
Sucrose	519.3	Trehalose	4.4
Phenolic acids			
	ng/mL		ng/mL
Homovanillic acid	12,091.49	ferulic acid	355.57
Vanillic acid	4771.12	*p*-hydroxobenzoic acid	314.21
Cinnamic acid	4469.30	Rosmarinic acid	191.40
Syringic acid	957.85	Chlorogenic acid	88.78
Sinapic acid	824.65	Gallic acid	18.40
Caffeic acid	681.80	3,4-dihydroxobenzoic acid	19.22
Benzoic acid	725.95	Salicylic acid	11.82
Coumaric acid	644.19	Gentisic acid	1.04
Phytohormones			
	pg/mL		pg/mL
t-zeatin	–	N6-(2-isopentenyl) adenine	124.4
c-zeatin	–	Kinetin riboside	33.6
kinetin	–	Indole-3-acetic acid	1418.3
t-zeatin riboside	–	Abscisic acid	9669.9
c-zeatin riboside	–	Indole-3-butyric acid	476.9

Pineapple pulp (PP) was far more abundant in sugars, phenolic acids, and phytohormones than was agar hydrolyzate (Table 2). It contained over 36 mg mL^−1^ of sugars, predominantly fructose, glucose, and maltose but also sucrose, kestose, isomaltotriose, nystose, and trehalose (Table 2). The content of phenolic acids in 1 mL of pineapple pulp reached 0.026 mg mL^−1^. Sixteen phenolic acids were found in PP, and homovanillic acid was the most common. Also, vanillic and cinnamic acids were found in high concentrations (Table 2). Total concentration of phytohormones in PP was 0.01 μg mL^−1^. Abscisic acid constituted 82 % of total concentration of phytohormones (9669.9 pg mL^−1^). Pineapple pulp contained also 1895.2 pg mL^−1^ of auxins and 158 pg mL^−1^ of cytokinins. The most abundant auxin was indole acetic acid (IAA), and the most abundant cytokinin was isopentenyladenine (iP).

### Micropropagation

**Fig. 1:**
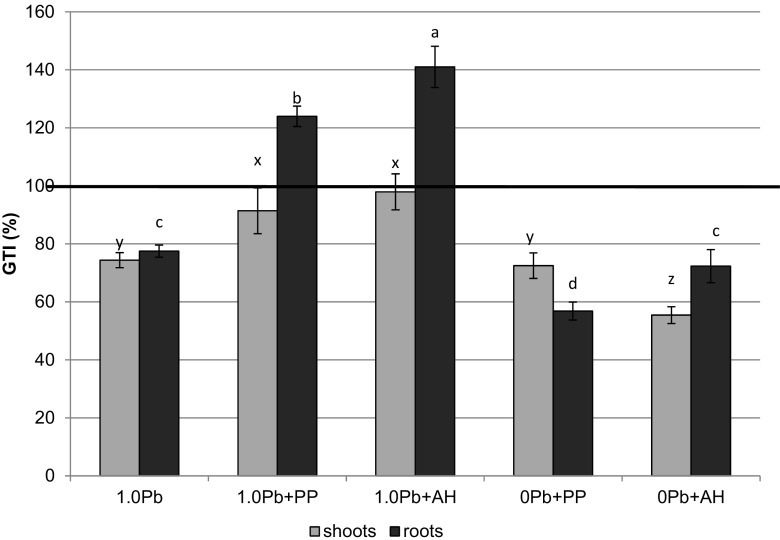
Growth tolerance index (GTI) for *Daphne jasminea* shoots and roots developed in the presence of Pb and organic supplements

Proliferative cultures of flowering and rooting shoots were obtained regardless of lead treatment, and complete microplantlets were developed. The efficiency of *D. jasminea* micropropagation on non-supplemented medium containing lead nitrate was comparable to that observed on the control medium without lead ions. Micropropagation coefficient (MC) was 7.8 and 8.1, respectively (*P* > 0.05) (Table 3). However, the growth of new shoots on Pb-containing medium was significantly inhibited, as expressed by the growth tolerance index for shoots (GTI_S_) of 74.3 % (Fig. 1) and reduced mean shoot height (Table 3). Several rooting characteristics decreased in lead-treated cultures, i.e., rooting percentage, GTI_R_ (77.4 %), and root length (Table 3). Number of roots per explant and dry weight of roots did not differ between Pb vs. non-Pb treatment (Table 3).

**Table 3:** Effectiveness of *Daphne jasminea* micropropagation after 16 weeks on media containing lead (II) nitrate and organic medium supplements

Treatment		MC^1^	Shoot length (mm)	Shoot dry weight (% fw)	Rooted shoots (%)	No. roots/microplant	Root length (mm)	Root dry weight (% fw)
Lead nitrate (mM)	Organic supplement							
1.0	–	7.8 ± 0.2b^a^	27.1 ± 1.7b	15.2 ± 0.3c	68.6 ± 2.8b	4.0 ± 0.2b	21.2 ± 0.6c	11.1 ± 0.3d
1.0	PP	8.6 ± 0.4a	29.7 ± 3.2b	16.4 ± 0.5b	87.1 ± 3.5a	4.8 ± 0.2a	20.5 ± 1.4c	12.9 ± 0.2c
1.0	AH	9.2 ± 0.5a	37.3 ± 2.5a	16.9 ± 0.1b	91.8 ± 4.7a	4.3 ± 0.1b	28.1 ± 3.1a	15.1 ± 0.6b
0	–	8.1 ± 0.1b	38.8 ± 2.2a	16.7 ± 0.2b	90.5 ± 3.9a	3.8 ± 0.4b	23.2 ± 2.2b	11.4 ± 0.2d
0	PP	5.1 ± 0.1d	15.0 ± 1.6c	18.9 ± 0.4a	24.1 ± 3.2d	0.5 ± 0.0c	23.9 ± 2.4b	19.8 ± 0.6a
0	AH	6.2 ± 0.3c	16.0 ± 2.3c	18.4 ± 0.3a	43.3 ± 4.1c	0.7 ± 0.1c	24.3 ± 1.8b	22.6 ± 1.7a

Means indicated by the same letter within the columns do not significantly differ at *P* < 0.05 according to Duncan’s test

*MC* micropropagation coefficient, *PP* pineapple pulp, *AH* agar hydrolyzate

^a^Values are means of three replicates ± SE

Growth tolerance index for lead increased significantly on the media containing organic supplements in comparison with non-supplemented Pb-medium. GTI_S_ for shoots increased by 22.9 % in PP and 31.8 % in AH while GTI_R_ for roots rose by 60.1 % in PP and of 82.4 % in AH (Fig. 1). Micropropagation coefficient increased by 10.2–16.6 % (*P* < 0.05) in comparison with non-supplemented Pb medium (Table 3). Rooting was also more efficient in the supplemented media (Table 3). Medium supplementation with AH enhanced shoot and root length, dry weight of both shoots and roots, and rooting efficiency (Table 3). The addition of pineapple pulp slightly improved rooting and increased the number of roots/explants and dry biomass (Table 3).

Surprisingly, in Pb-free media, organic additives negatively affected multiplication and growth of microplantlets. Micropropagation coefficient and shoot length decreased significantly on the medium containing AH or PP only (Table 3). Additionally, rooting was strongly inhibited in terms of number of roots/explants and rooting rate (Table 3). Growth inhibition was more pronounced in the presence of pineapple pulp than agar hydrolyzate. However, the dry weight of shoots and roots was the highest in these two treatments (Table 3).

### Accumulation of lead in cultured microplantlets

**Fig. 2:**
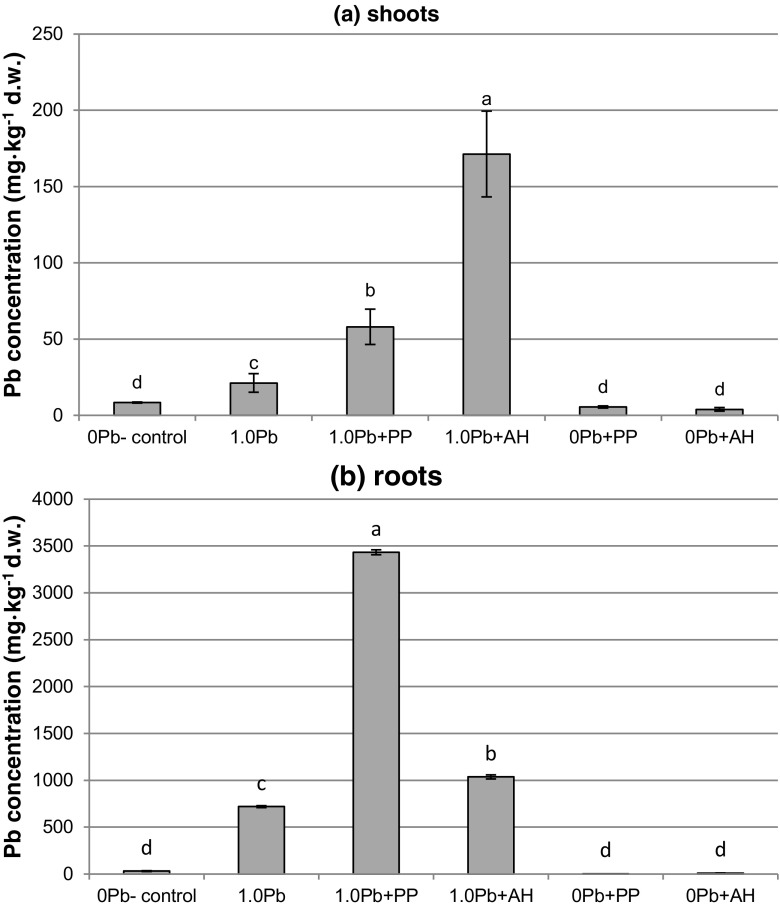
Lead accumulation in *D. jasminea* **a** shoots and **b** roots developed in the presence of Pb and organic supplements

*D. jasminea* microplantlets accumulated lead in both shoots and roots. However, significantly higher amounts of lead were detected in roots than in shoots (Fig. 2a, b). In the presence of organic supplements, lead accumulation increased significantly. In shoots, there was almost 3-fold increase of lead content in the tissues grown on the medium with PP and 8-fold increase in those grown on the medium with AH in comparison with non-supplemented medium (Fig. 2a). In roots, lead accumulation was 4.5- and 1.6-fold higher in PP- and AH-containing medium, respectively, than in non-supplemented one (Fig. 2b).

Considering bioconcentration factor (BCF) for lead, the highest value was calculated for PP-supplemented medium (BCF = 0.35) (Table 4). In the medium with agar hydrolyzate, BCF amounted to 0.12 and was slightly higher than in the medium without supplements (BCF = 0.07) (Table 4). Values of the translocation factor for lead were <1 for every treatment, indicating that lead was largely retained in the roots (Table 4). In the presence of agar hydrolyzate, TF increased significantly in comparison with other treatments (Table 4).

**Table 4:** Bioconcentration factor (BCF) and translocation factor (TF) for lead in *D. jasmine* after 16 weeks of culture in the presence of lead (II) nitrate and organic medium supplements

Treatment		Bioconcentration factor (BCF)	Translocation factor (TF)
Lead nitrate (mM)	Organic supplement		
1.0	–	0.07c	0.03b
1.0	PP	0.35a	0.02b
1.0	AH	0.12b	0.17a

Means indicated by the same letter within the columns do not significantly differ at *P* < 0.05 according to Duncan’s test

*PP* pineapple pulp, *AH* agar hydrolyzate

### Establishing a long-term culture (LT *Daphne* line) tolerant to lead

A long-term *D. jasminea* culture on the medium containing 1.0 mM of lead nitrate was established. The shoots multiplicated and rooted as efficiently as normal non-Pb-adapted shoots yielding microplantlets with the root system. The long-term culture was maintained for 1 year on the medium with lead and regular passages of 5-mm long shoot explants. Shoots from this stabilized long-term culture were used as a source of explants to conduct the experiment aimed at the comparison of some elements of the antioxidant system in Pb-tolerant and non-tolerant plants of *D. jasminea* in the presence of organic supplements.

### Phenolic compounds in microplantlets adapted and not adapted to Pb

#### Shoots

The shoots of LT line (adapted to lead) accumulated significantly higher amounts of phenolic compounds than did the shoots from LN line (non-adapted to lead). Depending on the treatment, the increase amounted to 2.5–5.0 times for total phenols, 3.4–7.6 times for phenylpropanoids, and 2–3.8 times for flavonols (Table 5). Only the concentrations of anthocyanins were lower in LT-shoots than in LN-shoots grown on the respective medium variants (Table 5). Considering LT-shoots exclusively, the application of organic supplements caused a decrease in the content of phenolic compounds. It was particularly clear in the medium with agar hydrolyzate (Table 5).

**Table 5:** Phenolic profile in Pb-adapted and non-Pb-adapted *D. jasminea* organs developed in the presence of Pb and organic supplements

Line/treatment	Total phenolics (mg CGA^1^/100 g fw)	Phenylpropanoids (mg CA^2^ /100 g fw)	Flavonols (mg QC^3^/100 g fw)	Anthocyanins (mg CY^4^/100 g fw)
Shoots				
LN/1.0Pb	622.48 ± 25.2d	183.39 ± 9.5d	202.08 ± 8.8d	44.44 ± 2.3a
LN/1.0Pb + PP^5^	702.02 ± 30.9d	232.62 ± 40.3d	262.51 ± 7.4c	38.24 ± 2.4ab
LN/1.0Pb + AH^6^	640.15 ± 3.8d	187.52 ± 19.3d	205.98 ± 12.7d	29.46 ± 3.1c
LT/1.0Pb	3008.84 ± 245.1a	1397.22 ± 36.1a	773.23 ± 29.5a	35.14 ± 2.3b
LT/1.0Pb + PP	2237.37 ± 106.2b	1028.56 ± 41.6b	567.90 ± 20.7b	37.73 ± 3.6ab
LT/1.0Pb + AH	1597.22 ± 143.1c	645.62 ± 49.2c	295.65 ± 15.3c	17.05 ± 3.1d
Roots				
LN/1.0Pb	191.92 ± 17.1e	30.44 ± 3.9e	29.89 ± 4.1f	2.58 ± 0.9d
LN/1.0Pb + PP	252.53 ± 12.2d	44.72 ± 1.7d	46.78 ± 3.9e	7.75 ± 1.6 cd
LN/1.0Pb + AH	282.83 ± 12.2d	43.59 ± 4.0d	61.08 ± 6.4d	10.85 ± 1.6c
LT/1.0Pb	2950.96 ± 142.1a	1353.25 ± 48.2a	690.71 ± 41.7a	37.72 ± 4.7a
LT/1.0Pb + PP	979.80 ± 64.1b	349.12 ± 30.0b	197.53 ± 14.1b	20.16 ± 6.2b
LT/1.0Pb + AH	560.961 ± 64.3c	218.71 ± 8.0c	130.60 ± 5.5c	5.43 ± 1.1 cd

Values represent means ± SE. For each organ, means followed by different letters within columns are significantly different at *P* < 0.05

*CGA* chlorogenic acid, *CA* caffeic acid, *QC* quercetin, *CY* cyanidin, *PP* pineapple pulp, *AH* agar hydrolyzate, *LN* line non-adapted to Pb, *LT* line adapted to Pb (long-term)

In LN-shoots (non-adapted to lead), total phenolic content and the concentration of phenylpropanoids were not affected by the addition of organic supplements to Pb-enriched medium. The content of flavonols was higher in the medium containing pineapple pulp, while the content of anthocyanins was the lowest in both supplemented media (Table 5).

#### Roots

Accumulation of phenolic compounds was more intensive in LT-roots than in LN-roots. Depending on the treatment, the level of total phenolics in LT-roots was higher from 2 to15 times, phenylpropanoids from 4 to 45 times, flavonols from 2 to 23 times, and anthocyanins from 2 to 14 times than in LN-roots developed on respective media (Table 5). The differences between both *Daphne* lines were particularly huge under lead treatment in the non-supplemented medium. Application of the medium supplements increased the content of phenolic compounds in LN-roots and decreased it in LT-roots (Table 5).

For the majority of treatments, the level of phenolic compounds in the roots was significantly lower than in the shoots. An exception was the culture of LT-line in the non-supplemented medium, where phenolic content in the roots was as high as in the shoots (Table 5).

### Radical scavenging activity in microplantlets adapted and not adapted to Pb

**Fig. 3:**
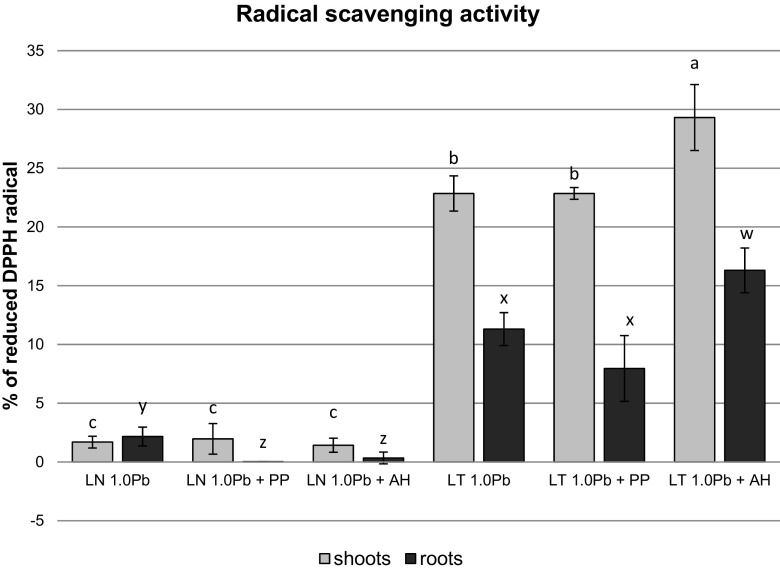
DPPH free radical scavenging activity in Pb-adapted and non-Pb-adapted *D. jasminea* organs developed in the presence of Pb and organic supplements

Radical scavenging activity was elevated in both shoots and roots of LT line in comparison with LN-organs. In fact, antiradical activity in the plants of non-adapted line was very low, with the efficiency of DPPH radical scavenging ranging between 1.42 and 1.96 % in the shoots and between 0.03 and 2.17 % in the roots (Fig. 3). In contrast, radical scavenging activity in LT-shoots ranged between 22.85 and 29.31 % and was significantly higher than in LT-roots (7.96–16.31 %). In LT-line, DPPH scavenging activity significantly increased in the presence of agar hydrolyzate (Fig. 3). Medium supplementation impaired antiradical activity in the roots of LN-line but had no effect on the shoots (Fig. 3).

## Discussion

An interesting feature of *D. jasminea* is its capability for effective in vitro proliferation, even in the presence of elevated concentration of lead ions (Noshad et al. 2009, Wiszniewska et al. 2013; Wiszniewska et al. 2015). In this study, we obtained branched microplantlets with root system, regardless of lead treatment. Micropropagation on Pb-containing medium was just as efficient as on the control medium, although growth tolerance of both shoots and roots decreased in the presence of lead nitrate. Growth inhibition is often associated with heavy metal-induced stress in cultured organs, particularly in the root system (Fernandez et al. 2008; Di Lonardo et al. 2011; Giampaoli et al. 2012). In our *D. jasminea* cultures, the negative effect of lead ions on root elongation and biomass was also visible. Despite that, the possibility of obtaining viable and rooted microplantlets may indicate a certain level of constitutional Pb tolerance in this *Daphne* species (Bojarczuk 2004; Wiszniewska et al. 2015).

Supplementation of the culture medium with organic additives advanced significantly the growth and tolerance of Pb-treated *D. jasminea* microplantlets. The increase covered commercially important characteristics, such as micropropagation coefficient and rooting percentage. The rise in the dry weight of both shoots and roots was related to enhanced biomass production. This, in turn, may reflect improved tolerance of the cultured organs to heavy metal stress, as reported in the cultures of tobacco, poplar, and Indian mustard (Nehnevajova et al. 2007; Rout and Sahoo 2007; Di Lonardo et al. 2011). In our study, the medium additives induced growth and stress tolerance of the root system that was particularly exposed to lead toxicity. Enhanced growth response could be attributed to the supplementation of nutrients and regulatory compounds that ameliorated adverse growth conditions and reduced unfavorable effects of lead presence. In fact, both supplements contained considerable amounts of carbohydrates, constituting an additional source of carbon and energy supply, as well as of plant growth regulators. Additional supplementation enabled biomass increase and undisturbed development of adventitious buds and roots that produced entire plantlets in suboptimal growth conditions. Both additives contained cytokinins and auxins but in different proportions. Exogenous application of phytohormones, either auxins or cytokinins, was reported to promote growth in plants under heavy metal stress (Meng et al. 2009; Piotrowska-Niczyporuk et al. 2012; Gemrotová et al. 2013). Here, phytohormones, exogenously supplied in organic additives, could counteract growth inhibition by balancing the level of growth regulators required for organogenesis. Moreover, pineapple pulp contained considerable amounts of abscisic acid. This agent plays an important role in the amelioration of growth response under unfavorable conditions caused by heavy metals (Hsu and Kao 2003; Wang et al. 2013).

In contrast, in the absence of Pb ions, *D. jasminea* responded negatively to the medium supplementation with organic additives. Organogenesis, multiplication, and growth were substantially inhibited. This was rather surprising, considering the composition of both additives: carbohydrates and cytokinins were expected to enhance biomass production, while phenolic compounds and auxins should improve rooting characteristics. The inhibitory effect can be explained by disturbed hormonal balance in the media containing organic additives. High concentration of abscisic acid in pineapple pulp could be responsible for the inhibitory effect of this medium supplement.

Interestingly, our analysis revealed that agar hydrolyzate contained small doses of various phytohormones. The agar tested here contained mainly cytokinins, while Arthur et al. (2004) reported on the presence of auxin-like compounds in their agar preparations. This indicates that agar may be an additional source of regulatory compounds in in vitro cultures. Agar types may contain different growth regulators, and thus differently affect the morphogenic fate of cultured plant tissues and organs. This is a noteworthy information considering optimization of medium composition in plant tissue culture.

Lead accumulation was noticed in *D. jasminea* culture, particularly in the roots. Very low values of the translocation factors suggest that majority of accumulated Pb was immobilized in the root system, protecting the aboveground parts from lead toxicity. This is the most common defense strategy of non-hyperaccumulating plants (Wierzbicka et al. 2007; Pourrut et al. 2013). In the presence of the medium additives, Pb accumulation in the roots and shoots was considerably enhanced. In this respect, pineapple pulp was superior to agar hydrolyzate. This could be due to different compositions of the tested supplements. Our study revealed that pineapple pulp contained considerable amounts of phenolic acids (0.026 mg mL^−1^), which is in agreement with other studies on chemical composition of pineapple residues (de Oliveira et al. 2009). Phenolic acids could improve Pb accumulation in *D. jasminea*, as natural polyphenols, e.g., phenolic acids and lignin, possess some bioremedial properties related to metal chelating, absorption, or coagulation (Kuppusamy et al. 2015). This has been recently reported for cadmium ions (Stingu et al. 2012; Li et al. 2015). Endogenous polyphenols were found to chelate Pb ions facilitating their accumulation in epidermal glands of water lily (Lavid et al. 2001). To the best of our knowledge, there are no other reports available on the effect of exogenous application of phenolic acids on Pb bioaccumulation in plants.

Higher accumulation of lead observed in the presence of pineapple pulp can also be attributed to higher concentration (over 200 times) of phytohormones, particularly auxins, than in agar hydrolyzate. There are numerous reports on improved heavy metal uptake and accumulation in plant organs by exogenously applied auxins (Fässler et al. 2010; Hadi et al. 2010; Vamerali et al. 2011; Hadi et al. 2013). In *Arabidopsis thaliana*, exogenous auxins promoted Cd binding to root cell walls, increasing metal concentration in roots (Zhu et al. 2013). Considering the influence of abscisic acid in pineapple pulp variant, our results seem to be in contrary to those obtained in *Populus × canescens* exposed to zinc (Shi et al. 2015) and *Arabidopsis* exposed to cadmium (Fan et al. 2014). In these reports, exogenous application of abscisic acid alleviated heavy metal stress by reduced accumulation of toxic ions. The opposite effect observed in *D. jasminea* exposed to Pb may be due to multiple interactions between exogenously applied phytohormones and phenolic acids in the culture medium, promoting Pb accumulation in the roots. Nevertheless, in this study, the bioconcentration factor was low (below 1), suggesting that *D. jasminea* plantlets were not efficient phytoextractors of lead. This is not surprising considering the low mobility of this metal. However, our results indicated that the bioaccumulation rate of lead may be increased by medium supplementation with pineapple pulp, even in the case of poorly accumulating plant species. Apart from the low bioaccumulation of Pb, its translocation to the aboveground parts of *D. jasminea* plants was limited as well. However, it was improved in the presence of agar hydrolyzate. Currently, we can only speculate on the mechanisms responsible for this phenomenon. The most plausible one is that agar hydrolyzate supplied the plantlets with substances, for example, phytohormones, which promote Pb transportation. Vodnik et al. (1999) reported on increased translocation of Pb ions to the shoots of Norway spruce in the presence of exogenously applied zeatin. The similar effect could occur in our study, as zeatin riboside was the most abundant phytohormone in agar hydrolyzate. Another possibility is the involvement of oligosaccharins, cell wall-derived bioactive molecules. However, as oligosaccharins failed to affect Cd distribution and accumulation (Kučerova et al. 2014), their role in enhanced transportation of Pb, which is a far less mobile element, is even less probable. Thus, further studies are required to look into this interesting phenomenon.

The study succeeded in establishing the long-term culture (LT-line) in Pb-containing medium in which shoots proliferated and formed adventitious roots. We used this culture to compare phenolic profile and radical scavenging activity between Pb-adapted (LT-line) and non-Pb-adapted (LN-line) plants in the presence of the investigated medium supplements. Generally, the level of phenolic compounds and radical scavenging activity were markedly higher in LT-organs than in LN-line. These differences suggested that Pb-adapted plants developed a more efficient antioxidant system during long culturing in the presence of Pb ions. Overproduction of phenolic compounds in *D. jasminea* LT-line may be associated with its increased tolerance to toxic ions, as this is one of the defense strategies against heavy metal stress (Kováčik and Bačkor 2007; Kováčik and Klejdus 2008). It is also considered a metabolic adaptation to elevated concentration of toxic metals (Barocsi et al. 2003; Wang et al. 2011a). As reported by Ali and Hadi (2015), elevated concentration of endogenous phenolics positively correlated with dry biomass accumulation in *Parthenium hysterophorus* exposed to cadmium, and thus contributed to enhanced growth tolerance in stress conditions.

The effect the medium supplementation exerted on the level of phenolic compounds was different for *D. jasminea* lines. In non-Pb-adapted shoots, it remained unchanged but it increased in the roots. However, the increase was not associated with an enhancement of antiradical activity of the roots. Therefore, higher concentration of phenolics may reflect a stress response of the roots triggered by the presence of the organic supplement. In Pb-adapted plants, concentration of phenolics markedly decreased, particularly in the roots. Simultaneously, antiradical activity in LT-line organs was as high in the medium with pineapple pulp as in the non-supplemented medium, and even higher in the medium with agar hydrolyzate. These responses may indicate that in the supplemented media, substances other than phenolics are involved in radical removal. Most likely, the activity and components of antioxidant machinery were differently regulated due to medium supplementation (Singh et al. 2004).

To conclude, the growth response of *D. jasminea* maintained in vitro on Pb-containing medium may be ameliorated by the addition of organic supplements. The supplements facilitated propagation of microplantlets and decreased their susceptibility to toxic ions. The study demonstrated that *D. jasminea* was not an efficient accumulator of lead; however, it utilized the mechanisms allowing for its tolerance and adaptation to the elevated level of this metal. Importantly, Pb accumulation may be enhanced by the addition of pineapple pulp or agar hydrolyzate, while lead translocation may be facilitated by using agar hydrolyzate. Our results indicated that the exploitation of organic amendments may be beneficial in in vitro selection against Pb toxicity. The presented approach may be useful in the research on revitalization of urban areas and also as an alternative to enhance phytoremediation. However, it is necessary to investigate the composition of the applied amendments and standardize them in order to expand their use in field-scale projects. Additionally, our study showed an inevitable advantage of using organic amendments, i.e., amelioration of plant growth in vitro in contaminated environment. Future research should focus on the evaluation of organic supplement usefulness in enhancing the tolerance in ex vitro conditions. Also, further investigation into the mode of action of organic supplements in alleviation of Pb toxicity would be valuable.
